# Secular trends of blood isolates in patients from a rural area population hospitalized in a tertiary center in a small city in Greece

**DOI:** 10.1186/1471-2180-6-41

**Published:** 2006-05-02

**Authors:** Matthew E Falagas, Alexandra Bakossi, Vasilis D Pappas, Pierros V Holevas, Antonis Bouras, Eleni Stamata

**Affiliations:** 1 Alfa Institute of Biomedical Sciences (AIBS), Athens, Greece; 2 Department of Medicine, Tufts University School of Medicine, Boston, Massachusetts, USA; 3 Department of Microbiology, General Hospital of Tripolis, Tripolis, Peloponnesus, Greece

## Abstract

**Background:**

Most of the studies evaluating the secular trends of blood isolates come from tertiary hospitals in urban areas. We sought to study the trends of the antimicrobial resistance of blood isolates in patients from a rural population hospitalized in a tertiary hospital in a small city in Greece.

**Methods:**

We retrospectively collected and analysed data for the first positive blood culture obtained for each admission for each patient hospitalized in General Hospital of Tripolis, Tripolis, Peloponnesus, Greece during a 5 year period (16/05/2000 – 15/05/2005).

**Results:**

Sixty-seven thousand and seventy patients were hospitalized during the study period from whom 3,206 blood cultures were obtained. A higher increase of the number of obtained blood cultures than the number of admissions was noted during the study period (p < 0.001). Three hundred and seventy-three (11.6%) blood cultures were positive. Coagulase-negative staphylococci (35.9%), *Escherichia coli *(29%), and *Staphylococcus aureus *(18.2%) were the most commonly isolated pathogens. Among the *Staphylococcus aureus *isolates, the proportion of methicillin-resistant *Staphylococcus aureus *(MRSA) was 17.2% (5/29). The proportion of *Escherichia coli *resistant to trimethoprim and sulfamethoxazole, ampicillin and cefuroxime was 29.6% (32/108), 25.0% (27/108), and 8.3% (9/108) respectively. Imipenem-resistance was noted in 3.4% (1/29) of *Pseudomonas aeruginosa *isolates. There were only 6 (1.6%) *Acinetobacter baummanii *blood isolates during the study period.

**Conclusion:**

The antimicrobial resistance of isolates from patients receiving care at the studied tertiary hospital in a small city in Greece is considerably less compared to that noted in tertiary hospitals in larger cities of the country.

## Background

There is an increasing incidence of multi drug-resistant (MDR) bacterial infections in several parts of the world [1]. However, the majority of the studies on the secular trends of antimicrobial resistance have been performed in tertiary medical centers in large cities. There is relatively scarcity of information regarding the trends of antimicrobial resistance in patients from rural populations hospitalized in healthcare centers in small cities. Thus, we sought to collect and analyze data relevant to this significant public health problem from the General Hospital of Tripolis, a tertiary center serving a rural area of Peloponnesus, Greece.

## Methods

### Patient population

The patient population consisted of patients admitted to the General Hospital of Tripolis, Tripolis, Greece, during the period of 16/05/2000 – 15/05/2005. The General Hospital of Tripolis is a 204-bed, tertiary health center that offers services of most medical specialties, including medicine, surgery, pediatrics, and obstetrics and gynecology. The hospital serves the population of the city of Tripolis (40,000 people) and the population of the surrounding rural area of about 250,000 people (local, mainly rural population of Central and South Peloponnesus, Greece). Our study did not involve any experimentation on patients. It is a retrospective analysis of microbiological data. The study was approved by the Department of Microbiology of General Hospital of Tripolis, Greece and by the Ethics Committee of the Alfa Institute of Biomedical Sciences, Athens, Greece.

### Microbiological studies

In our study, isolates of the first positive blood cultures obtained for each admission for each patient were analyzed. The isolation of the microorganisms from blood culture specimens was performed using the Bact/Alert 3D 60 Select (Biomerieux) automated system, according to the manufacturer's instructions. We focused on Gram-negative and Gram-positive bacterial isolates. Identification of the microorganisms to the species level was performed with the API system (Biomerieux Vitek, Hazelwood, MO). Gram-negative microorganisms were identified by the API 20 E strip system, a self-contained system of 20 microtubes of dehydrated substrates designed for overnight incubation. Identification is performed by adding necessary reagents and then visually interpreting the results. The tests included in the system, are: o-nitrophenyl galactopyranoside (ONPG), arginine dehydrolase (ADC), lysine and ornithine decarboxylase (LDC and ODC respectively), citrate (CIT), H_2_S, urea (URE), tryptophan deaminase (TDA), indole (IND), Veges-Proscone (VP), gelatin (GEL), glucose (GLU), mannitol (MAN), inositol (INO), sorbitol (SOR), rhamnose (RHA), sucrose (SAC), melibiose (MEL), amygdalin (AMY), and arabinose (ARA). Numerical coding of results allows computerized interpretation of patterns, lists of which are available in a codebook. Streptococci were identified with the API 20 strep system. *Staphylococcus aureus *was differentiated from other catalase-positive, Gram-positive cocci by mannitol salt agar. Pseudomonas species were identified with the use of the oxidase test. The fungi isolates were Candida but they were not further identified to the species level.

Bacterial isolation was followed by antimicrobial susceptibility testing that was performed using the disc diffusion in Mueller-Hinton agar and measurement of the diameter of the inhibition zones technique. The antibiotic discs that we used were provided by MAST Diagnostics, Mast Group Ltd, Bootle, Merseyside, United Kingdom. The diameters of microbial inhibition were interpreted based on the relevant guidelines regarding the cutoff diameters of the Clinical and Laboratory Standards Institute (CLSI, formerly called NCCLS) for each antibiotic tested, depending on the microbial species (NCCLS volume 21-1, M2-A7 document). In our study we included data regarding the in vitro susceptibility of blood isolates to antimicrobial agents only if they were available for the whole 5-year study period, except for results of in vitro susceptibility testing of *S. aureus *and coagulase negative staphylococci to oxacillin and penicillin.

### Statistical analysis

Differences in proportions were compared by x^2 ^test or Fischer exact test if the numbers of the studied was small (less than 6 in any of the compared cells of observations). The statistical significance was set at the level p < 0.05. All statistical analyses were performed using SPSS 11.0 (SPSS Inc., Chicago, Illinois, USA).

## Results

During the study period, 67,070 patients were hospitalized at the General Hospital of Tripolis and 3,206 blood cultures were performed. The annual number of admissions in the hospital, the number of blood cultures performed, and the proportion of positive cultures per year are presented in Table 1. A gradual increase of the number of admissions and the number of blood cultures was noted during the study period (p < 0.001). Compared to data of the first year of the study a 14.1% and 122.7% increase of the number of admissions and the number of performed blood cultures, respectively, was noted during the fifth year of the study. On the other hand the proportion of positive blood cultures did not significantly increase (p = 0.39) resulting in bloodstream infection rates remaining stable over the 5-year period (between 9.9%–13.3%).

Data regarding the isolated microorganisms from the first positive blood culture for each patient for each admission during the study period are presented in Table 2. Gram-positive bacteria were the most common isolates. Coagulase-negative staphylococci comprised 35.9% of the total isolates. Following, in descending order, *Escherichia coli *comprised 29.0%, *S. aureus *18.2%, *Pseudomonas aeruginosa *7.8%, *Acinetobacter baumannii *1.6%,*Proteus mirabilis *1.6%, and *Klebsiella pneumoniae *1.1%. The statistical analysis did not yield significant changes in the proportion of isolates of different species during the 5-year period. The analysis of the relative frequency of blood isolates per year showed that *E. coli *comprised the commonest isolate during the first and second year of the study while it became the second commonest after coagulase-negative staphylococci during the last 3 years of the study (p = 0.01). The p-values for the comparison of the frequency of isolates of different species during the study period were 0.28, 0.06, and 0.45 for coagulase-negative staphylococci, *E. coli *and *S. aureus*, respectively. *A. baumannii *blood isolates were reported for the first time during the third year of the study.

The results of the analysis of the available data of the in vitro antimicrobial testing of blood isolates during the study period are presented in Table 3. The proportion of methicillin-resistant *S. aureus *(MRSA) was 17.2% (5/29); no in vitro antimicrobial susceptibility testing of staphylococcal isolates to oxacillin was performed during the last 2 years of the study due to the unavailability of the relevant discs with the antibiotic. The proportion of *E. coli *resistant to trimethoprim and sulfamethoxazole, ampicillin, and cefuroxime was 29.6% (32/108), 25.0% (27/108), and 8.3% (9/108) respectively. The proportion of *E. coli *isolates resistant to trimethoprim and sulfamethoxazole did not alter during the study period (p = 0.71). Imipenem-resistance was noted in 3.4% (1/29) of *Pseudomonas aeruginosa *isolates. There were only 6 (1.6%) *Acinetobacter baummanii *blood isolates during the study period. An imipenem-resistant *A. baumanni *strain was isolated during the fifth year of the study.

## Discussion

Most of the studies that report increased trends in resistance to antimicrobial agents have been performed in tertiary urban hospitals. Comparisons of reported data of nosocomial infections among hospitals of different bed sizes show that the risk of developing nosocomial infection and hospital-acquired bacteremia is directly associated with the size of each hospital [2-4]. In addition, another observation is that MDR strains are more commonly isolated in large than in small hospitals [3,5,6]. Our study suggests that another factor besides the hospital size that is associated with the proportion of MDR isolates may be the location of a hospital, i.e. whether the health care facility is in a large or a small city. This may be partially explained by differences in the morbidity of the average patient and antibiotic prescription policies of hospitals of same size in large and small cities. However, another epidemiologic reason leading to differences of antimicrobial resistance of isolates of patients who receive care in hospitals in large and small cities may be the fact that patients are more likely to be transferred from one hospital to another in large cities. This is especially true in countries such as Greece where transfer of patients between hospitals is not uncommon, for example transfer from a private to a public hospital or the reverse.

An interesting finding of our analysis concerns the disproportionate increase of the number of obtained blood cultures compared to the increase of the number of admissions during the study period. Annual increase in the number of performed blood cultures is a finding that has been reported by investigators in other studies as well [7,8]. We consider that this is partly attributed to therapeutic policies adherent to current guidelines that suggest obtaining blood cultures in various patient populations [9].

The major isolates cultured in our study, were coagulase negative staphylococci, *E. coli*, and *S. aureus*. Totally, Gram-positive bacterial isolates were more common, than Gram-negative. These findings are in keeping with results reported from other hospitals [10]. It is noteworthy that while during the first study period gram-negative bloodstream infection was more pronounced, gram-positive bacteremia prevailed during the last period, defining in this way a shift in the causative pathogens [11].

The commonest type of blood isolate during the study period was coagulase negative staphylococci. This may be misleading, as a great proportion of coagulase negative staphylococci isolates obtained from blood cultures, are contaminants and not true agents of blood stream infection [12,13]. The inclusion of each patient's first positive blood culture only, comprises a limitation to the interpretation of these data. However, coagulase negative staphylococci, once isolated, should be always taken into account as an agent potentially responsible for bacteremia in severely ill patients, when we decide about the therapeutic regimen [14,15].

The number of blood cultures yielding Candida isolates, also increased during the study period. Such an increase is reported in other studies as well and is attributed to many factors, including the vigorous use of broad-spectrum antibiotics, the increased length of survival of immunocompromised patients, the presence of indwelling catheters [16,17].

Regarding the trends in resistance patterns of isolated pathogens to different antimicrobial regimens, an increasing trend of the resistance of isolated *P. aeruginosa *strains to amikacin, ceftazidime and imipenem was noted during the last period of our study. An optimistic observation was the fact that MDR *A. baumanii *was uncommonly isolated in the hospital of our study [18-20].

## Conclusion

In summary, we found that the antimicrobial resistance of isolates from patients receiving care at the studied tertiary hospital in a small city in Greece is considerably less compared to that noted in a study of ours using similar methodology in a tertiary hospital in a larger city (Athens) of our country [21].

## Competing interests

The author(s) declare that they have no competing interests.

## Authors' contributions

MEF had the idea for the study and supervised it. MEF and VDP wrote the first draft of the manuscript. ASB, PVH, AB, and ES collected the data. All authors made revisions of the manuscript and approved its final version.

## Figures and Tables

**Table 1:** Number of admissions and total and positive blood cultures during each study period in General Hospital of Tripolis, Greece (16/05/2000–15/05/2005).

	**Hospital admission period**						
	**2001**	**2002**	**2003**	**2004**	**2005**	**2001–2005 (total)**	**p-value**
Hospitalized patients	12,625	12,655	13,559	13,835	14,396	67,070	< 0.001
Number of blood cultures obtained	406	483	650	763	904	3,206	< 0.001
Ratio of admissions to blood cultures obtained	31:1	26:1	21:1	18:1	16:1	21:1	
Positive blood cultures (proportion from blood cultures obtained)	46 (11.3)	48 (9.9)	71 (10.9)	88 (11.5)	120 (13.3)	373 (11.6)	0.39

**Table 2:** Relative frequency of blood isolates in General Hospital of Tripolis, Greece (2001–2005).

	Number of isolates (proportion) within the specified periods*						
**Microorganisms**	**2001**	**2002**	**2003**	**2004**	**2005**	**Total**	**p-value**

**Gram-positive**							
*Coagulase-negative staphylococci*	15 (32.6)	15 (31.3)	26 (36.6)	26 (29.5)	52 (43.3)	134 (35.9)	0.28
*Stapylococcus aureus*	6 (13)	9 (18.8)	14 (19.7)	21 (23.9)	18 (15)	68 (18.2)	0.45
*Streptococcus viridans*			1 (1.4)			1 (0.3)	
**Gram-negative**							
*Escerichia coli*	20 (43.5)	17 (35.4)	22 (31)	22 (25)	27 (22.5)	108 (29)	0.06
*Pseudomonas aeruginosa*	2 (4.3)	3 (6.3)	4 (5.6)	11 (12.5)	9 (7.5)	29 (7.8)	
*Acinetobacter baumannii*			3 (4.2)	2 (2.3)	1 (0.8)	6 (1.6)	
*Proteus mirabilis*	1 (2.2)	3 (6.3)	0(0)	0(0)	2 (1.6)	6 (1.6)	
*Klebsiella pneumoniae*	1 (2.2)	1 (2.1)	0(0)	0(0)	2 (1.6)	4 (1.1)	
*Enterobacter*			1 (1.4)			1 (0.3)	
**Candida spp**	1 (2.2)			4 (4.6)	7 (5.8)	12 (3.2)	
**Miscellaneous Gram-negative**							
*Serratia marscescens*					1 (0.8)	1 (0.3)	
*Morganella morgannii*				1 (1.1)		1 (0.3)	
*Citrobacter*				1 (1.1)		1 (0.3)	
*Achromobacter*					1 (0.8)	1 (0.3)	
*TOTAL*	46 (100)	48 (100)	71 (100)	88 (100)	120 (100)	373 (100)	

* for the isolate of the first positive blood culture

**Table 3:** Trends of antimicrobial resistance of Gram-positive and Gram-negative bacteria in General Hospital of Tripolis, Greece (2001–2005).

	**Number of resistant isolates/total isolates (proportion) within the year**					
***Microorganisms***	**2001**	**2002**	**2003**	**2004**	**2005**	**Total**

***Gram-positive***						
***Staphylococcus epidermidis***						
Oxacillin*	0/15 (0)	6/15 (40)	7/26 (26.9)			13/56 (23.2)
Gentamicin	2/15 (13.3)	1/15 (6.7)	1/26 (3.8)	0/26 (0)	9/52 (17.3)	13/134 (9.7)
Vancomycin	0/15 (0)	0/15 (0)	0/26(0)	0/26 (0)	0/52 (0)	0/134 (0)
Penicillin				13/26 (50)	40/52 (76.9)	53/78 (67.9)
***Staphylococcus aureus***						
Oxacillin*	0/6 (0)	5/9 (55.6)	0/14 (0)			5/29 (17.2)
Gentamicin	1/6 (16.7)	1/9 (11.1)	0/14 (0)	3/21 (14.3)	1/18 (5.6)	6/68 (8.8)
Vancomycin	0/6 (0)	0/9 (0)	0/14 (0)	0/21 (0)	0/18 (0)	0/68 (0)
Penicillin				14/21 (66.7)	13/18 (72.2)	27/39 (69)
***Streptococcus viridans***						
Penicillin			0/1 (0)			0/1 (0)
Vancomycin			0/1 (0)			0/1 (0)
***Gram-negative***						
***Escherichia coli***						
Trimethoprim-sulfamethoxazole	8/20 (40)	6/17 (35.2)	6/22 (27.2)	6/22 (27.2)	6/27 (22.2)	32/108 (29.6)
Amikacin	8/20 (40)	4/17 (23.5)	1/22 (4.5)	0/22 (0)	6/27 (22.2)	19/108 (17.6)
Gentamicin	2/20 (10)	4/17 (23.5)	0/22 (0)	0/22 (0)	2/27 (7.4)	8/108 (7.4)
Ampicillin	3/20 (15)	0/17 (0)	6/22 (27.2)	8/22 (36.3)	10/27 (37.0)	27/108 (25.0)
Cefuroxime	0/20 (0)	0/17 (0)	2/22 (9.1)	1/22 (4.5)	6/27 (22.2)	9/108 (8.3)
***Pseudomonas aeruginosa***						
Amikacin	0/2 (0)	0/3 (0)	0/4 (0)	0/11 (0)	3/9 (33.3)	3/29 (10.3)
Ceftazidime	0/2 (0)	0/3 (0)	0/4 (0)	2/11 (18.2)	2/9 (22.2)	4/29 (13.8)
Ciprofloxacin	1/2 (50)	0/3 (0)	0/4 (0)	4/11 (36.4)	3/9 (33.3)	8/29 (27.6)
Imipenem	0/2 (0)	0/3 (0)	0/4 (0)	0/11 (0)	1/9 (11.1)	1/29 (3.4)
***Acinetobacter baumanii***						
Amikacin			0/3 (0)	1/2 (50)	0/1 (0)	1/6 (16.7)
Gentamicin			0/3 (0)	0/2 (0)	0/1 (0)	0/6 (0)
Ceftazidime			3/3 (100)	1/2 (50)	0/1 (0)	4/6 (66.7)
Ciprofloxacin			3/3 (100)	2/2 (100)	0/1 (0)	5/6 (83.3)
Imipenem			0/3 (0)	0/2 (0)	1/1 (100)	0/6 (0)
***Klebsiella pneumoniae***						
Ceftazidime		0/1 (0)			0/2 (0)	0/3 (0)
Ciprofloxacin		0/1 (0)			1/2 (50)	1/3 (33.3)
Meropenem		0/1 (0)			0/2 (0)	0/3 (0)
Cefepime		0/1 (0)			0/2 (0)	0/3 (0)
Cefoxitin		1/1 (100)			0/2 (0)	1/3 (33.3)
Tobramycin		0/1 (0)			0/2 (0)	0/3 (0)

* Oxacillin discs were not available for antimicrobial testing during the period 16/05/2003 – 15/05/2005
